# Developmental Regulation of Protein O-GlcNAcylation, O-GlcNAc Transferase, and O-GlcNAcase in Mammalian Brain

**DOI:** 10.1371/journal.pone.0043724

**Published:** 2012-08-22

**Authors:** Ying Liu, Xiaojing Li, Yang Yu, Jianhua Shi, Zhihou Liang, Xiaoqin Run, Yi Li, Chun-ling Dai, Inge Grundke-Iqbal, Khalid Iqbal, Fei Liu, Cheng-Xin Gong

**Affiliations:** 1 Department of Neurochemistry, New York State Institute for Basic Research in Developmental Disabilities, Staten Island, New York, United States of America; 2 Jiangsu Key Laboratory of Neuroregeneration, Nantong University, Nantong, Jiangsu, China; Centre Hospitalier de l’Université Laval, Canada

## Abstract

O-GlcNAcylation is a common posttranslational modification of nucleocytoplasmic proteins by β-N-acetylglucosamine (GlcNAc). The dynamic addition and removal of O-GlcNAc groups to and from proteins are catalyzed by O-linked N-acetylglucosamine transferase (O-GlcNAc transferase, OGT) and β-N-acetylglucosaminidase (O-GlcNAcase, OGA), respectively. O-GlcNAcylation often modulates protein phosphorylation and regulates several cellular signaling and functions, especially in the brain. However, its developmental regulation is not well known. Here, we studied protein O-GlcNAcylation, OGT, and OGA in the rat brain at various ages from embryonic day 15 to the age of 2 years. We found a gradual decline of global protein O-GlcNAcylation during developmental stages and adulthood. This decline correlated positively to the total protein phosphorylation at serine residues, but not at threonine residues. The expression of OGT and OGA isoforms was regulated differently at various ages. Immunohistochemical studies revealed ubiquitous distribution of O-GlcNAcylation at all ages. Strong immunostaining of O-GlcNAc, OGT, and OGA was observed mostly in neuronal cell bodies and processes, further suggesting the role of O-GlcNAc modification of neuronal proteins in the brain. These studies provide fundamental knowledge of age-dependent protein modification by O-GlcNAc and will help guide future studies on the role of O-GlcNAcylation in the mammalian brain.

## Introduction

Protein O-GlcNAcylation is a unique type of protein glycosylation. It refers to the enzymatic transfer of β-N-acetylglucosamine (GlcNAc) from UDP-GlcNAc donor to the hydroxyl group of serine/threonine residues of proteins via an O-glycosidic bond [1]. This process is catalyzed by O-linked N-acetylglucosamine transferase, or O-GlcNAc transferase (OGT, E.C. 2.4.1.94). O-GlcNAc on proteins can also be removed with the catalysis of β-N-acetylglucosaminidase, or O-GlcNAcase (OGA, E.C. 3.2.1.52). Protein O-GlcNAcylation is dynamically regulated by these two enzymes.

Compared with classical O-linked glycosylation, O-GlcNAcylation has three key features: (i) It occurs in nucleocytoplasmic compartments rather than endoplasmic reticulum and Golgi. (ii) It is dynamic and analogous to protein phosphorylation, with cycling in response to cellular signals or cellular stages. (iii) All O-GlcNAcylated proteins that have been identified so far are also phosphoproteins. In some proteins, O-GlcNAcylation and phosphorylation competitively modify the same serine/threonine residues and are thus reciprocal to each other [2].

Many studies have demonstrated that O-GlcNAcylation is ubiquitous in viruses and all metazoans, including plants, worms, insects and mammals, and regulates many cellular signaling and functions, including transcription, translation, protein degradation, cell signaling, cell trafficking, apoptosis, and cell cycle control [2], [3]. In the central nervous system, numerous proteins are modified by O-GlcNAc. Our recent proteomic studies have identified O-GlcNAcylation of 274 proteins in the mouse brain [4]. These proteins play critical roles in various brain functions. O-GlcNAcylation has also been shown to regulate many synaptic proteins and neuronal cytoskeleton proteins [5], [6], [7], [8], [9], [10], [11], [12]. We recently found that a down-regulation of O-GlcNAcylation occurs in the brains of individuals with Alzheimer’s disease and contributes to neurofibrillary degeneration [7], [13], [14].

Selective OGT knockout that eliminates protein O-GlcNAcylation in neurons leads to neuronal apoptosis [15]. Systematic knockout of OGT results in loss of embryonic stem cell viability [16]. These observations suggest that O-GlcNAc might regulate neurodevelopment. However, the developmental regulation of O-GlcNAcylation and its catalytic enzymes, OGT and OGA, has not been well investigated. In this report, we fill this important gap of knowledge with a detailed demonstration of protein O-GlcNAcylation, OGT, and OGA in the rat brain during development from embryonic day 15 (E15d) to the age of 2 years (P2y).

## Materials and Methods

### Antibodies and Reagents

The primary antibodies used in this study are listed in Table 1. Peroxidase-conjugated anti-mouse and anti-rabbit IgG were obtained from Jackson ImmunoResearch Laboratories (West Grove, PA, USA). The enhanced chemiluminescence (ECL) kit was from Amersham Pharmacia (Piscataway, NJ, USA). Human recombinant nuclear pore glycoprotein p62 was purchased from ABcam (Cambridge, MA, USA). Polyethylene glycol 8000 was from Promega (Madison, WI, USA). Ecoscint A was from National Diagnostics (Atlanta, GA, USA). Other chemicals were from Sigma (St. Louis, MO, USA).

**Table 1:** Primary antibodies employed in this study.

Antibody	Type	Specificity	Source/Reference
RL2	Mono-	O-GlcNAc	Affinity Bioreagents, Golden, CO, USA
CTD110.6	Mono-	O-GlcNAc	Covance, Emeryville, CA, USA
Anti-OGT (TI-14)	Poly-	OGT	Sigma Aldrich, St. Louis, MO, USA
Anti-OGA	Poly-	OGA	[41]
Anti-pSer	Poly-	Phosphoserine	Invitrogen, Carlsbad, CA, USA
Anti-pThr	Poly-	Phosphothreonine	Invitrogen, Carlsbad, CA, USA

### Animals

Wistar rats were from Charles River Laboratories, Inc. (Wilmington, MA, USA). Pregnant female rats were sacrificed at 15 (E15d) and 19 (E19d) days of gestation, and the brains of rat fetuses (n = 10 for E15d and n = 6 for E19d) were dissected immediately. Rat brains were also collected from pups on the day of birth (P0), and male rats (n = 3–5/group) at postnatal day 5 (P5d), P15d, post-natal month 1 (P1m), P6m, post-natal year 1 (P1y), and P2y. All rats were sacrificed between 2 and 3 o’clock in the afternoon. The animal experiments were performed according to the “Principles of Laboratory Animal Care” (NIH Publication 86-23, revised in 1985) and were approved by the Animal Welfare Committee of the New York State Institute for Basic Research in Developmental Disabilities.

### Western Blot Analysis and Immuno-dot-blot Assays

Rat forebrain tissue was homogenized in buffer consisting of 50 mM Tris-HCl (pH 7.4), 2.0 mM EDTA, 10 mM β-mercaptoethanol, 8.0 µg/ml aprotinin, 100 µg/ml leupeptin, 4.0 µg/ml pepstatin, and 8.5% sucrose. Aliquots of the homogenates were mixed immediately by the same volume of 2× concentrated Laemmeli buffer (125 mM Tris-HCl, pH 6.8, 4% SDS, 20% glycerol, 2% β-mercaptoethanol, and 0.005% bromophenal blue), followed by heating in boiling water for 5 min. Protein concentrations of the samples were determined by using modified Lowry method [17]. The levels of global O-GlcNAcylation and phosphorylation as well as of OGT and OGA were determined by Western blots using 10% sodium dodecyl sulfate–polyacrylamide gel electrophoresis (SDS-PAGE). The Western blots were developed by using ECL, and the immunoreactivities of the blots were quantified densitometrically.

For immuno-dot-blot assays of global protein phosphorylation in the rat brain, the homogenates were sonicated for 10 min and centrifuged briefly. The supernatants were dotted onto nitrocellulose membrane and developed with anti-phosphoserine and anti-phosphothroenine antibodies, as described previously [18].

### OGT and OGA Activity Assays

For OGT assay, the rat brain homogenates were first centrifuged at 16,000 *g* at 4°C for 10 min, and the resulting extracts were mixed with 2 volumes of cold 30% polyethylene glycol 8000 (dissolved in 25 mM HEPES containing 10 mM MgCl_2_, pH 7.23) to precipitate proteins. The samples were then centrifuged at 16,000 *g* again to remove salts as supernatants. The protein pellets were resuspended in the OGT assay buffer (25 mM HEPES, pH 7.5, 10 mM MgCl_2_ and 1 mM EDTA) and centrifuged at 16000 *g* at 4°C for 20 sec. The resulting supernatants (i.e., desalted extracts) were then incubated at 24°C for 2 h in the OGT assay buffer that also included 10 µM [^3^H]UDP-GlcNAc (800 µCi/pmol), 8.0 µg/ml p62. The reactions (50 µl each) were stopped by adding 100 µl of cold 15% trichloroacetic acid. After cooling in wet ice for 10 min, the mixtures were centrifuged at 16,000 *g* for 30 sec, and the pellets were washed with 10% trichloroacetic acid twice. The final pellets were resuspended in 150 µl of 0.5% SDS, followed by bath sonication for 5 min. The dissolved proteins were finally transferred into scintillation vials and mixed with Ecoscint A for scintillation counting of the radioactivity transferred on p62. Aliquots of the same desalted extract samples were also assayed in the presence of 5 mM alloxan, an OGT inhibitor [19], and the radioactive differences between the presence and the absence of alloxan were used for calculation of OGT activities.

OGA activities of the brain samples were determined by using a method modified from Gao et al. [20]. Briefly, the crude rat brain extracts were incubated in the assay buffer (100 µl/assay) containing 50 mM sodium cacodylate (pH 6.4), 2 mM p-nitrophenyl-N-acetyl-β-D-glucosaminide, 0.3% bovine serum albumin, 50 mM GalNAc, 2 mM β-mercaptoethanol, 2 mM EDTA, 1 mM AEBSF, 5 µg/ml leupepetin, 4 µg/ml pepstatin, and 8 µg/ml aprotinin at 37°C for 30 min. Then the reactions were stopped with the addition of 0.9 ml of 0.5 M sodium carbonate, and absorbance at 400 nm was measured.

### Immunohistochemistry

Tissue was first fixed in 10% PBS-buffered formalin and embedded in paraffin. Thin (6-µm-thick) sections were cut, and the sections were washed thoroughly and permeabilized with 0.4% Triton X-100 in PBS for 90 min. Nonspecific binding was blocked with normal goat serum for 2 h, followed by incubation with primary antibody (1∶200) at 4°C overnight. The immunoreactivity was developed by using biotinylated goat-anti rabbit or goat anti-mouse IgG (1∶200), the avidin-biotin complex (VECTASTAIN® ABC kits, Vector Labs, Inc., Burlingame, CA), and 3,3′-diaminobenzidine (DAB) staining. The immuno-stained sections were examined by using an Optiphot-2 (Nikon) light microscope. All images of stained sections were digitized under the same illumination and diaphragm aperture settings.

### Statistical Analyses

Where appropriate, the data are presented as mean ± SD, and ANOVA was used for calculation of the statistical significance among groups. For analysis of the correlation between global protein phosphorylation and global protein O-GlcNAcylation, the Pearson correlation coefficient *r* was calculated.

## Results

### Overall Brain Protein O-GlcNAcylation Declines during Development

**Figure 1:**
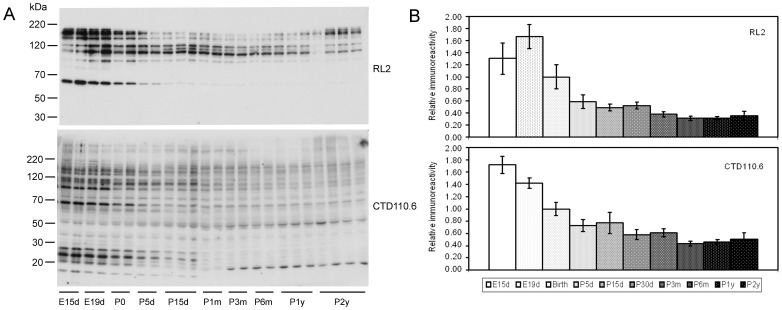
Developmental changes of protein O-GlcNAcylation in the rat brain. (**A**) Equal amounts of brain homogenates from rats of various ages were analyzed by Western blots developed with antibody RL2 and CTD110.6 to detect O-GlcNAcylated proteins. (**B**) The blots were quantified densitometrically, and the values of each age point were plotted against ages. The data are presented as relative immunoreactivity (mean ± SD, n = 3–10), and the mean values at birth (P0) were defined as 1.0. The age-dependent changes of both RL2 and CTD110.6 immunoreactivity were statistically significant (p<0.05 by ANOVA).

To investigate the developmental changes of protein O-GlcNAcylation, we analyzed O-GlcNAcylated proteins in homogenates from brains of rats with different ages by using Western blots developed with two antibodies that recognize O-GlcNAcylated proteins. These two antibodies are raised by using two different O-GlcNAcylated immunogens (nuclear pore complex-lamina fraction from rat liver for RL2 and a synthetic peptide containing serine-O-GlcNAc for CTD110.6) and are known to recognize the majority of O-GlcNAcylated proteins, although each antibody has different affinities to different O-GlcNAcylated proteins. We found that both antibodies stained multiple protein bands of brain samples obtained at various ages (Fig. 1A). Because the same amount of homogenate proteins (10 µg/lane) from each sample was loaded for SDS-PAGE, we were able to quantify the global O-GlcNAcylation by densitometry of the blots. We found that the total brain protein O-GlcNAcylation decreased dramatically during early development (from E15d through P5d) and then declined slightly during late development (from P5d through P3m) (Fig. 1B). The total protein O-GlcNAcylation level in the brain remained unchanged after maturation of rats till 2 years.

A comparison of the RL2 blots with the CTD110.6 blots indicates that antibody CTD110.6 recognized a larger number of O-GlcNAcylated proteins than RL2 in the rat brain (Fig. 1A). CTD110.6 stained numerous brain proteins of apparent molecular weights ranging from larger than 220 kD to smaller than 20 kDa, whereas RL2 did not stain any brain proteins of ∼65 kDa or smaller. Except for the ∼20-kDa and 50-kDa proteins seen in the CTD110.6 blots, O-GlcNAcylation of most proteins declined with a similar pattern during early development. The proteins with the most dramatic decline in their O-GlcNAcylation included those of an apparent molecular weight of ∼68 kDa and between 20 and 30 kDa. Taken together, these results indicate a high level of protein O-GlcNAcylation during early brain development.

### Various OGT Isoforms are Differentially Regulated in the Brain during Development

**Figure 2:**
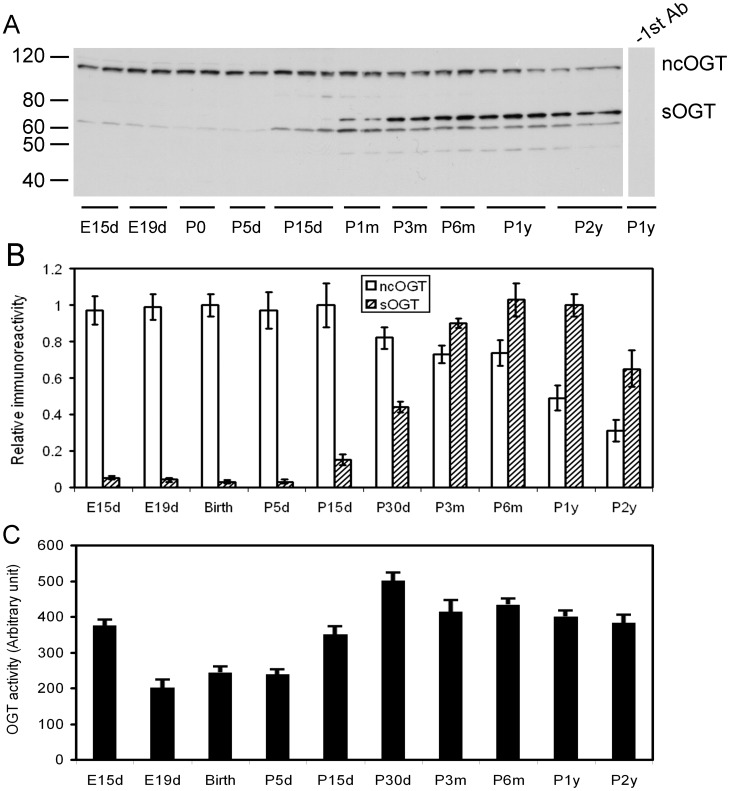
Level and activity of OGT in the rat brain during development. (**A**) Equal amounts of brain homogenates from rats of various ages were analyzed by Western blots developed with antibody against OGT. As a control, anti-OGT was omitted during incubation with the primary antibody solution for the lane at right side. (**B**) The ncOGT band and sOGT bands of the blots were quantified separately by densitometry, and the mean values of each age point were plotted. The data are presented as relative immunoreactivity (mean ± SD, n = 3–10), and the mean values at birth (P0, ncOGT) or at P1y (sOGT) were defined as 1.0. (**C**) The OGT activity (mean ± SD, n = 3–10) was determined toward p62 as a substrate. The age-dependent changes of ncOGT and sOGT levels and the OGT activity were statistically significant (p<0.05 by ANOVA).

The *OGT* gene on the X chromosome is capable of producing three separate transcripts, and each encodes a different OGT isoform: nucleocytoplasmic OGT (ncOGT, 116 kDa), mitochondrial OGT (mOGT, 103 kDa), and the shortest form of OGT (sOGT, 70 kDa) [21]. Western blots developed with a polyclonal antibody against OGT revealed a marked and stable expression of ncOGT in the rat brain during development (Fig. 2A,B). The ncOGT level decreased gradually after 1 month and reduced by up to 60% when rats were 2 years old. In contrast, the brain sOGT level was nearly undetectable during early development and increased markedly after P15d, although its level decreased again at 2 years. Two positive bands at ∼70 kDa were seen. Whether there are two sOGT variants in the rat brain, or one of these two bands resulted from cross-reaction remains to be elucidated. In addition, there was another weak immuno-positive band of ∼50 kDa, the level of which altered similarly to that of sOGT during development. The identity of this weak band is presently unknown. We did not observe significant expression of mOGT in the rat brain, except that a very weak band of mOGT was sometimes seen at the 103 kDa position in P15d to P1m rats.

We also measured the total OGT activity toward p62, a commonly used substrate for OGT assays, in the crude rat brain extracts and found a three-phase alteration of OGT activity during development. The OGT activity was high at E15d and declined by nearly half at E19d (Fig. 2c). The brain OGT activity kept low levels from E19d to P5d and then increased till P1m, followed by a small decline till P2y.

**Figure 3:**
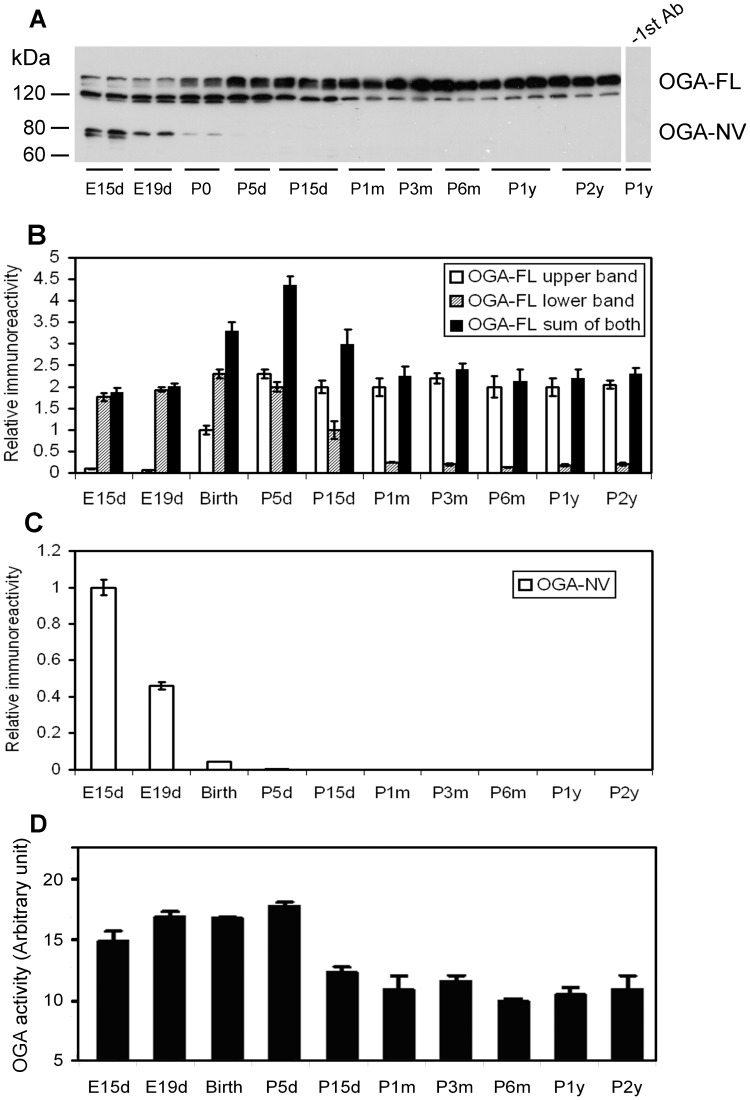
Level and activity of OGA in the rat brain during development. (**A**) Equal amounts of brain homogenates from rats of various ages were analyzed by Western blots developed with antibody against OGA. As a control, anti-OGT was omitted during incubation with the primary antibody solution for the lane at right side. (**B**) The upper and lower bands of OGT-FL of the blots were quantified individually by densitometry, and the mean values of each age point were plotted. The data are presented as relative immunoreactivity (mean ± SD, n = 3–10), and the mean value of the OGA-FL upper band at birth (P0) was defined as 1.0. (**C**) The OGA-NV bands of the blots were also quantified densitometrically, and the mean ± SD values (n = 3–10) of each age point were plotted. (**D**) The OGA activity in the brain extracts was determined toward p-nitrophenyl-N-acetyl-β-D-glucosaminide as a substrate. The age-dependent changes of the levels and activity of OGA were statistically significant (p<0.05 by ANOVA).

### OGA-FL and OGA-NA are Differentially Regulated in the Brain during Development

Two splice variants of OGA are encoded from a single gene. The cytosolic full-length (OGA-FL) variant has a predicted MW of 103 kDa and an apparent MW of 120–130 kDa. The nuclear variant (OGA-NV) lacks a putative histone acetyl transferase C-terminal domain and has an apparent molecular weight of 75 kDa [22]. Western blots developed with a polyclonal antibody against OGA revealed a 130-kDa band and a 120-kDa band that both appear to be OGT-FL (Fig. 3A), although we could not completely rule out the possibility that one of these two immuno-positive bands results from a cross-reaction of the antibody we used. The upper OGA-FL band was expressed at a low level during early development (E15d–E19d) and then at increasing levels from P0 to P5d, after which age the upper OGA-FL level kept unchanged till P2y. In contrast, the level of the lower OGA-FL band was high during early development (E15d–P5d) and then decreased from P5d to P1m, after which age the 120-kDa OGA-FL level stayed at a low level till P2y. When we quantified both the upper and the lower OGA-FL bands, the total level peaked at P5d and remained unchanged during adulthood (Fig. 3B). The OGA-NV level declined rapidly during early development and was undetectable after birth (Fig. 1A,C).

**Figure 4:**
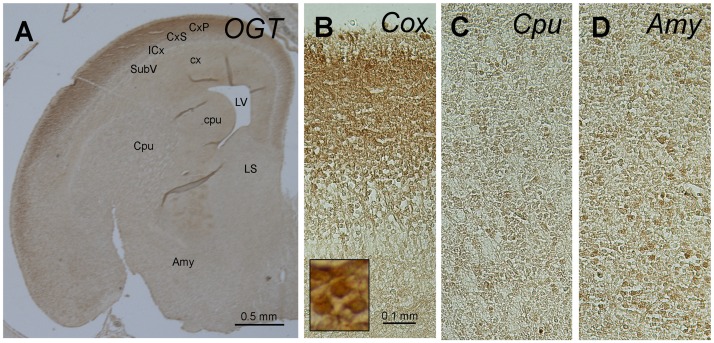
Immunohistochemical staining of E19d rat brain sections with antibody against OGT. Abbreviations: Amy, amygdala; Cox, cerebral cortex; Cpu, caudate putamen; cpu, caudate/putamen neuroepithelium; CxP, cortical plate; CxS, cortical subplate; ICx, intermediate cortical layer; LV, lateral ventricle; SubV, subventricular cortical layer.

Each of the OGA bands was actually composed of at least two closely migrated bands (Fig. 3A), probably due to posttranslational modifications of OGA in the rat brain.

The OGA activity of rat brain was found to be high during early development from E15d to P5d (Fig. 3D). The activity dropped by 40–50% during the rest of the lifespan (P15d to P2y). The age-associated changes of OGA activity resembled those of the total level of OGA-FL plus OGA-NV (Fig. 3B,C).

**Figure 5:**
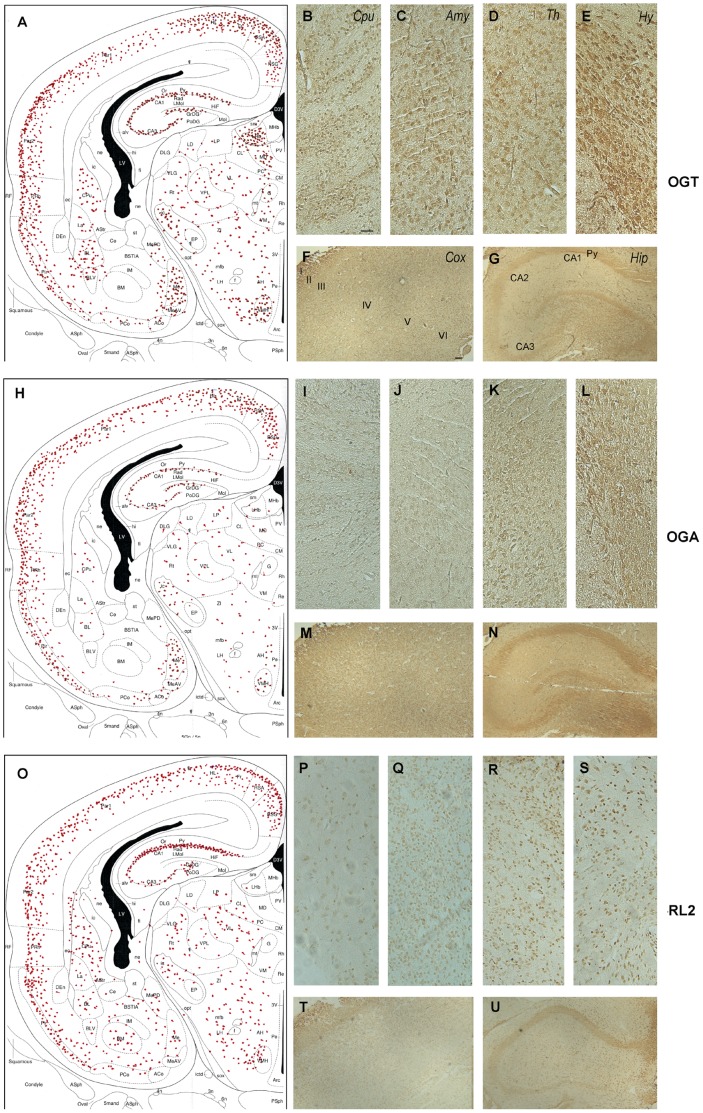
Immunohistochemical staining of P5d rat brain sections with antibodies against OGT (A–G), OGA (H–N) and O-GlcNAc (P–U). Panels A, H and O are illustrations that represent the density of the immunostaining observed in various regions of the brain. Abbreviations: Amy, amygdala; CA cornu ammonis; Cox, cerebral cortex; Cpu, caudate putamen; Hip, hippocampus, Hy, hypothalamus; Py, pyramidal neurons, Th, thalamus. Scale bars  = 500 µm.

### Immunohistochemical Distribution of O-GlcNAcylated Proteins, OGT, and OGA in Rat Brains at Various Ages

We investigated the topographic distribution of O-GlcNAcylated proteins, OGT, and OGA during development by immunohistochemistry. For these studies, we selected four time points (E19d, P5d, P6m, and P1y) to cover embryonic and postnatal development as well as the adult stages. We found nearly identical distribution of O-GlcNAcylated proteins (data not shown), OGT (Fig. 4), and OGA (data not shown) in the E19d rat fetal brains. The strongest immuno-staining was seen in the cortical plate (CxP), whereas the staining in cortical subplate (CxS), intermediate cortical layer (ICx), and subventricular cortical layer (SubV) was much weaker and similar to that in the other areas of the fetal brain. At higher magnification, we observed higher intensity of immunostaining in the cytoplasm than in the nucleus of most neurons, but both cell compartments were stained (Fig. 4, insert). As a control for staining specificity, we also included brain sections in the identical procedure except the omission of the primary antibody. No positive staining was seen under these conditions (data not shown), suggesting that the immunostaining we observed resulted from the immunoreactions with the primary antibody.

**Figure 6:**
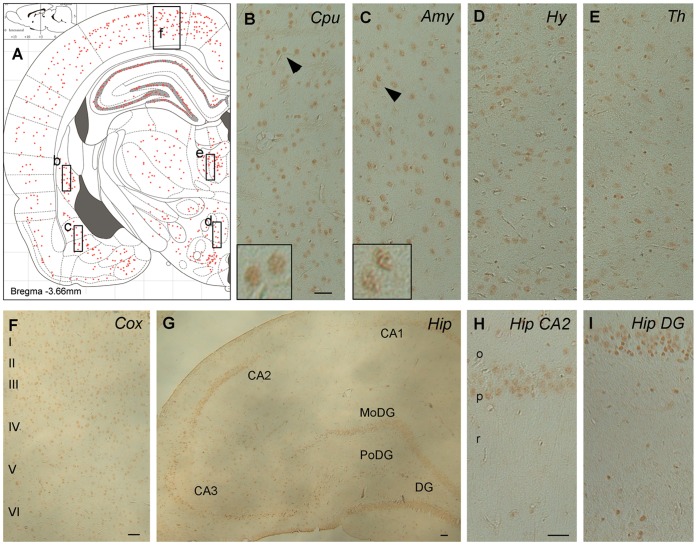
Immunohistochemical staining of P6m rat brain sections with O-GlcNAc antibody CTD110.6. Panel A is an illustration that represents the density of the immunostaining observed in various regions of the brain. Black arrowheads in panels B and C indicate the neurons shown in the inserts. Abbreviations: Amy, amygdala; CA, cornu Ammonis; Cox, cerebral cortex; Cpu, caudate putamen; DG, dentate gyrus; MoDG, molecular dentate gyrus; PoDG, polymorph dentate gyrus; Hip, hippocampus, Hy, hypothalamus; o, stratum oriens; p, pyramidal neurons; r, stratum radiatum; Th, thalamus. Scale bars  = 50 µm.

At postnatal day 5, the overall staining patterns of OGT, OGA, and O-GlcNAcylated proteins (stained both with RL2 and CTD110.6) were similar, except weaker staining for OGA was seen in the subcortical areas (Fig. 5). Both neuronal cell bodies and cell processes were stained. The topographic distribution of the whole hemisphere evaluated from intensity of the immunostaining is diagrammatically illustrated in Fig. 5A, 5H, and 5O. Laminar immunostaining was seen in the cortex, especially with anti-OGT. Positive neuronal staining of OGT and O-GlcNAc was also very intense in several subcortical nuclei such as the amygdala, thalamus, and hypothalamus (Fig. 5A–E, 5O–S). OGA staining in the subcortical areas was much weaker (Fig. 5H–L). The pyramidal neurons of the hippocampus were moderately stained for OGT, OGA and O-GlcNAc, but the granule neurons of the dentate gyrus were much more weakly stained with OGT and OGA and were not stained with O-GlcNAc (Fig. 5G,N,U).

**Figure 7:**
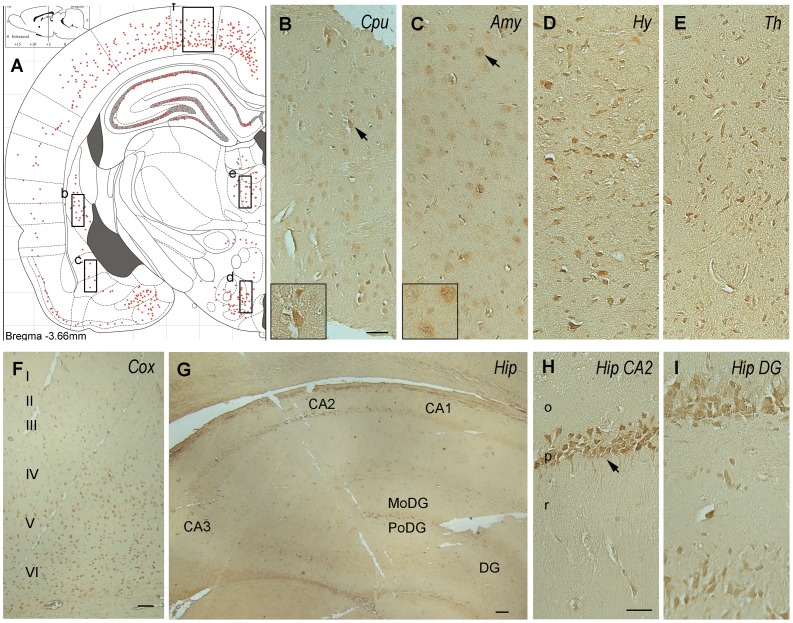
Immunohistochemical staining of P6m rat brain sections with anti-OGT. Panel A is an illustration that represents the density of the immunostaining observed in various regions of the brain. Abbreviations: Amy, amygdala; CA, cornu ammonis; Cox, cerebral cortex; Cpu, caudate putamen; DG, dentate gyrus; MoDG, molecular dentate gyrus; PoDG, polymorph dentate gyrus; Hip, hippocampus, Hy, hypothalamus; o, stratum oriens; p, pyramidal neurons; r, stratum radiatum; Th, thalamus. Scale bars  = 50 µm.

**Figure 8:**
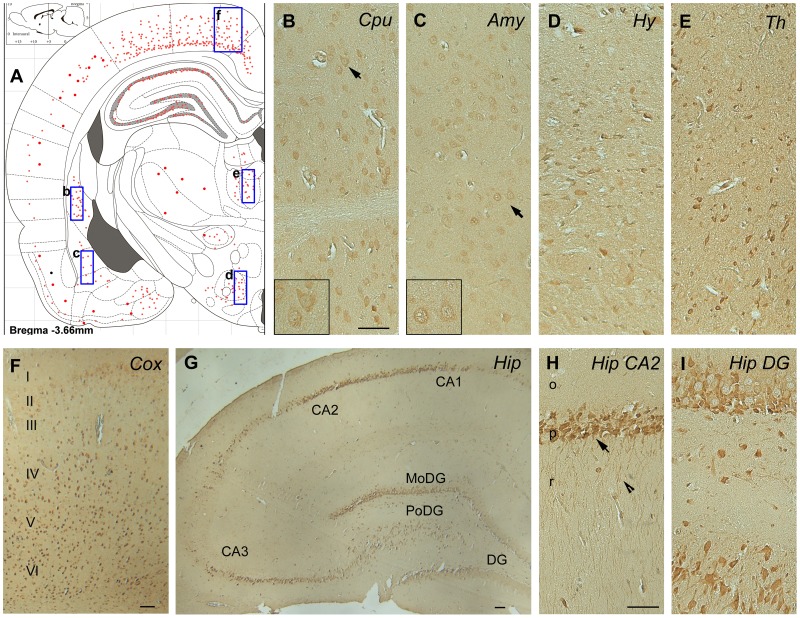
Immunohistochemical staining of P6m rat brain sections with anti-OGA. Panel A is an illustration that represents the density of the immunostaining observed in various regions of the brain. Black arrows in panels B and C indicate the neurons shown in the inserts. Abbreviations: Amy, amygdala; CA, cornu ammonis; Cox, cerebral cortex; Cpu, caudate putamen; DG, dentate gyrus; MoDG, molecular dentate gyrus; PoDG, polymorph dentate gyrus; Hip, hippocampus, Hy, hypothalamus; o, stratum oriens; p, pyramidal neurons; r, stratum radiatum; Th, thalamus. Scale bars  = 50 µm.

Antibodies CTD110.6 (Fig. 6) and RL2 (data not shown) to O-GlcNAcylated proteins stained numerous neurons throughout the brains of 6-month-old rats with almost identical topography. The staining was moderate in almost all brain structures, suggesting that O-GlcNAcylation modification is not region- or neuron-specific in the adult rat brain. In the hippocampus, both the pyramidal neurons of the cornu Ammonis and the granule neurons of the dentate gyrus were moderately stained (Fig. 6G–I). Both nuclear and cytoplasmic staining was seen at a higher magnification (Fig. 6B, C, inserts), which is consistent with the nucleocytoplasmic distribution of O-GlcNAcylation. The OGT (Fig. 7) and OGA (Fig. 8) staining also had nearly identical distributions in the 6-month-old rat brain. Moderate to strong staining was seen in the dorsal parietal cortex and some sectors of the hippocampus. Only moderate staining was seen in the rest of the cerebral cortex and in the caudate putamen (Figs. 7B, 8B), amygdala (Figs. 7C, 8C), hypothalamus (Figs. 7D, 8D), and thalamus (Figs. 7E, 8E), whereas very weak neuronal staining was seen in the rest of the 6-month-old rat brain. In the hippocampus, the pyramidal neurons in the CA3 sector were not stained with either OGT or OGA, whereas the pyramidal neurons in the rest of the hippocampus were stained moderately to strongly (Figs. 7G, 8G). High magnification indicated both nuclear and cytoplasmic staining for OGT (Fig. 7B,C, inserts) and more cytoplasmic than nuclear staining for OGA in the majority of neurons (Fig. 8B,C, inserts). Less nuclear staining of OGA is consistent with the lack of OGA-NV in the rat brain after birth, as determined by Western blots (Fig. 3A,C).

The overall staining patterns of O-GlcNAcylated proteins, OGT, and OGA in the brains of 1-year-old rats were similar to those at 6 months old (data not shown), suggesting no marked changes in the topography of O-GlcNAcylated proteins, OGT, or OGA.

**Figure 9:**
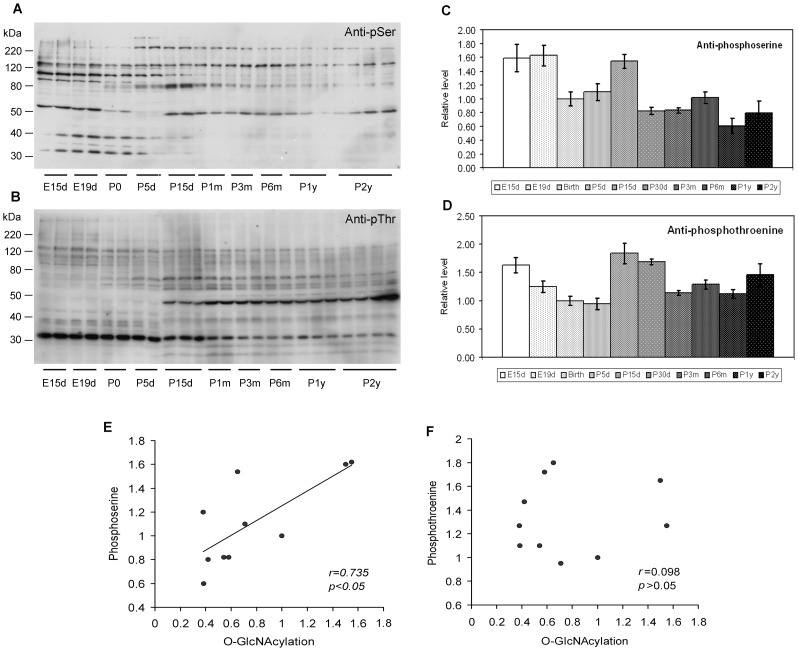
Levels of protein phosphorylation and their correlation with those of O-GlcNAcylation of rat brain proteins at various ages. Equal amounts of brain homogenates from rats of various ages were analyzed by Western blots developed with antibody anti-phosphoserine (anti-pSer) (**A**) or anti-phosphothreonine (anti-pThr) (**B**). The levels of total phosphoserine (**C**) and phosphothreonine (**D**) in the rat brain homogenates were measured by using immuno-dot-blot assays. The mean ± SD values (n = 3–10) of each age point were plotted. The data represent relative levels, and the mean values at birth (P0) were defined as 1.0. The age-dependent changes of the immunoreactivities with anti-pSer and anti-pThr were statistically significant (p<0.05 by ANOVA). Correlation analysis between global protein O-GlcNAcylation, measured by quantification of all positive bands in the RL2 and CTD110.6 blots, and global protein phosphoserine (**E**) or phosphothroenine (**F**), measured by immuno-dot-blot assays, was also performed. A linear regression line is shown in E because the correlation reached statistical significance.

### Correlation between Global O-GlcNAcylation and Global Phosphorylation of Proteins in the Rat Brain

O-GlcNAcylation often regulates phosphorylation of proteins and, in some cases, these two posttranslational modifications regulate each other in a reciprocal manner. To investigate whether the age-associated decline of global O-GlcNAcylation in the rat brain (Fig. 1) is associated with an increase of global protein phosphorylation, we analyzed global protein phosphorylation by Western blots developed with anti-phosphoserine (Fig. 9A) and anti-phosphothreonine (Fig. 9B). As expected, both antibodies stained multiple protein bands in the rat brain, and the immunoreactivity of most of these proteins altered during development. We also quantified the global protein phosphorylation level in the rat brains by using immuno-dot-blot assays and found variant levels of protein phosphorylation at serine (using anti-phosphoserine) and threonine (using anti-phosphothreonine) residues at different ages. The overall brain protein phosphorylation at serine residues was found to be much higher during embryonic stages and at P15d than other ages (Fig. 9C). The overall brain protein phosphorylation at threonine residues was high at E15d and declined during early development till P5d (Fig. 9D). At the age of P15d, its level was elevated again and then declined gradually during the rest of life. Correlation analysis revealed a weak, but significant, positive relationship between global protein phosphorylation at serine residues and global protein O-GlcNAcylation (*r* = 0.736, *p*<0.05; Fig. 9E), but no significant relationship between global protein phosphorylation at threonine residues and global protein O-GlcNAcylation (Fig. 9F). These results suggest a lack of overall reciprocal relationship between protein O-GlcNAcylation and phosphorylation in the rat brain throughout development.

## Discussion

To date, nearly one thousand nucleocytoplasmic proteins have been found to be modified by O-GlcNAc. It has been demonstrated that O-GlcNAcylation regulates multiple functions of the cell. Earlier observations showing that OGT knockout is lethal for the embryo [16] indicate that protein O-GlcNAcylation is essential for life. Neuronal apoptosis occurs after selective OGT knockout [15], suggesting that O-GlcNAcylation might regulate neurodevelopment. However, the developmental regulation of O-GlcNAcylation and its catalytic enzymes, OGT and OGA, in the brain have not been well investigated. The present study has filled this important gap of knowledge. We found that the level of global brain protein O-GlcNAcylation was high at the early developmental stage, and it gradually decreased during development. The changes in the total level of protein O-GlcNAcylation through the lifespan correlated positively to the changes of total protein phosphorylation at serine residues, but did not correlate significantly to those of total protein phosphorylation at threonine residues in the rat brain. These observations also revealed a lack of overall reciprocal regulation between O-GlcNAcylation and phosphorylation of rat brain proteins at the global level. The expression of different isoforms of OGT and OGA was regulated differently at different ages of rats. Immunohistochemical studies revealed that O-GlcNAcylation was ubiquitous and was seen throughout the brain at all ages. Strong immunostaining of O-GlcNAc, OGT, and OGA was observed mostly in neuronal cell bodies and processes, further suggesting the role of O-GlcNAc modification of neuronal proteins in the brain.

Because the O-GlcNAcylation was detected by antibodies against O-GlcNAcylated proteins in the present study, some of the changes may come from changes in the levels of proteins varying during development. Thus, the different levels of protein O-GlcNAcylation we observed in the present study represented the sum of the levels of O-GlcNAcylated proteins and the O-GlcNAc modification in the brain. Protein O-GlcNAcylation is regulated by circadian rhythm [23]. All rats were sacrificed and the brain samples were collected in the early afternoon, which eliminated the effect of circadian rhythm on our study.

Fülöp *et al.* [24] compared the total levels of brain O-GlcNAc and OGT of rats between 5 and 24 months old and found only a slight difference between these two age points. These observations are consistent with our findings that dramatic changes of protein O-GlcNAcylation and OGT were seen only during early development and before maturation of the animals. By using ELISA developed with antibody RL2, Rex-Mathes *et al*. [25] studied O-GlcNAc levels in the brains of 3- to 13-month-old mice and found a higher level of brain O-GlcNAc at 5 months of age than other ages. However, we did not find any marked changes in the level of brain O-GlcNAc between 3 and 13 months old. Whether the discrepancy between these two studies is due to two different species (mouse vs. rat) remains elusive.

We observed a differential expression of ncOGT and sOGT in the rat brain. During maturation, the level of ncOGT gradually declined, while that of sOGT increased markedly. These observations suggest a differential role of ncOGt and sOGT in the brain at different stages of life. Two separate bands at ∼70 kDa were stained by anti-OGT in Western blots, suggesting that some sOGT might contain posttranslational modifications in the brain, although the possibility that one of the two bands might result from antibody cross-reaction cannot be ruled out. Phosphorylation and auto-O-GlcNAcylation of OGT have been reported previously [26], [27], [28]. An important question in the O-GlcNAcylation field is how a single enzyme can act on a vast array of tissues and on many different protein substrates in a regulated fashion. Part of the answer might be that it is regulated by modifications and is associated with different partners. We did not observe mOGT in the rat brain at any ages, except a very weak band at the mOGT position was seen at the ages of P15d to P1m. Because the OGT antibody used in this study recognized both ncOGT and sOGT, it is unlikely that this antibody did not recognize mOGT if it were present in the rat brain. It has been reported previously that the mitochondrial isoform of OGT has the most limited tissue expression (http://genome.ucsc.edu/cgi-bin/hgNear).

The *OGA* gene encodes two isoforms of the enzyme as the result of alternative splicing of its messenger RNA: the cytosolic full-length OGA, and the smaller nuclear variant [22]. We observed that the full-length OGA is expressed in the rat brain throughout the whole lifespan, whereas the nuclear variant was highly expressed during early development, and its level became undetectable after birth. It is interesting to note that the anti-OGA stained two protein bands at the 120–130 kDa position, with the upper protein band increasing and the lower band decreasing during development (Fig. 3A). The complementary changes in the levels of the two protein bands during development and adulthood suggest that the 120-kDa band could be a truncated form of the 130-kDa OGA. Alternatively, both protein bands can be the full-length OGA with different levels of posttranslational modifications, such as phosphorylation, that affect the gel mobility. OGA has been reported to be modified by phosphorylation at Ser364 [29], Thr709 [30], Ser859, Ser867, and Ser873 [31] and to be O-GlcNAcylated at Ser405 [9]. Similar patterns between the age-dependent alterations of the total OGA activity in the brain extracts and the alterations of the sum of the 120-kDa and 13-kDa bands, but not of either individual band, further supports that both of these bands represent active OGA. However, we cannot completely rule out the possibility that one of these two immuno-positive bands results from a cross-reaction of the antibody we used.

The ubiquitous distribution of O-GlcNAc, OGT, and OGA in the rat brain is consistent with the involvement of protein O-GlcNAcylation in multiple functions in the central nervous system, especially during the early stages of brain development when the global O-GlcNAcylation was found to be the highest. Both OGT and OGA are much more abundant in brain tissue than other tissue [20], [32]. Predominant neuronal staining of O-GlcNAc, OGT, and OGA over non-neuronal cells in the brain suggests the direct involvement of O-GlcNAcylation in neuronal regulation and functions. Previous observations of O-GlcNAc modifications of numerous neuronal proteins [4], [33] are consistent with the present study. O-GlcNAcylation has also been shown to regulate the entry of neurons into an axon branching program [34].

O-GlcNAcylation is a sensor of intracellular glucose metabolism, because the OGT activity is mainly regulated by the intracellular level of UDP-GlcNAc, which is a metabolic product of glucose through the hexosamine biosynthetic pathway [35], [36]. Our observation of higher protein O-GlcNAcylation level is in agreement with the higher metabolic rate at the earlier developmental stages of the brain. It is interesting to note that the gradual decline of global O-GlcNAcylation throughout the lifespan was not accompanied by a gradual decline of OGT or a gradual increase in OGA in the rat brain. These observations suggest that the overall protein O-GlcNAcylation in the brain may not be mainly regulated by the levels of the catalytic enzymes, but instead by other factors, such as intracellular UDP-GlcNAc.

Under certain conditions, protein O-GlcNAcylation and phosphorylation regulate each other in a reciprocal manner [7], [13], [37], [38], [39]. This reciprocal relationship was not seen at the global level in this study, because O-GlcNAcylation declined during development, but such a decline was not accompanied by an age-dependent increase in global protein phosphorylation. In contrast, the global O-GlcNAcylation was found to be correlated positively to the global phosphorylation at serine residues of brain proteins during development, but the positive correlation appeared to depend heavily on two data points at the far end of the plot (Fig. 9E). Thus, a reciprocal relationship between O-GlcNAcylation and phosphorylation may not be generalized. Consistent with the present study, Hart and his colleagues demonstrated a complex interplay between O-GlcNAcylation and phosphorylation, with a reduction of phosphorylation at 280 sites and an elevation of phosphorylation at 148 sites of 711 phosphopeptides after elevation of O-GlcNAcylation [40].

In summary, the present study has demonstrated, for the first time, detailed developmental regulation of global O-GlcNAcylation and its catalytic enzymes in mammalian brain from embryonic day 15 to 2 years of rat. These observations provide fundamental knowledge of age-dependent protein modification by O-GlcNAc and will help future studies on the roles of O-GlcNAcylation in the central nervous system at various ages.
